# Supplementary material for: The Interleukin-11/IL-11 Receptor Promotes Glioblastoma Survival and Invasion under Glucose-Starved Conditions through Enhanced Glutaminolysis
Source: Int J Mol Sci. 2023 Feb 8;24(4):3356. doi: 10.3390/ijms24043356 (PMC9960532; doi:10.3390/ijms24043356)
Supplement: Supplementary file 1 [file ijms-24-03356-s001.zip › ijms-2112254-supplementary.pdf]

International Journal of *Molecular Sciences*

Article

The Interleukin-11/IL-11 Receptor Promotes Glioblastoma Survival and Invasion under Glucose-Starved Conditions through Enhanced Glutaminolysis

Sarah F. Stuart [1,2], Ayenachew Bezawork-Geleta [3], Zammam Areeb [1], Juliana Gomez [1], Vanessa Tsui [1], Ahmad Zulkifli [1], Lucia Paradiso [1], Jordan Jones [1,4], Hong P. T. Nguyen [1], Tracy L. Putoczki [1,5,6], Paul V. Licciardi [7,8], George Kannourakis [2,9], Andrew P. Morokoff [1,4], Adrian A. Achuthan [10] and Rodney B. Luwor [1,2,9,*]

Supplementary Files

Supplementary Table S1. The Correlation of the Expression of the IL-6 cytokine family with Glioblastoma Patient Survival

Cytokine	Average survival of Patients with High Expression (Days; n = 38)	Average survival of Patients with Low Expression (Days; n = 38)	P value
IL-11	**350**	**566**	**0.018**
IL-6	378	465	0.169
IL-27	437	341	0.974
CNTF	448	381	0.353
LIF	357	499	0.101
OSM	409	478	0.277

Supplementary Table S2. The Correlation of IL-11Rα Expression in Primary Glioblastoma Cell lines with Survival of Patients of which Cell line was originally taken.

Cell Line	IL-11Rα Expression	Survival Days Post Diagnosis
#35	*High*	*112*
#41	High	*124*
#15	High	*142*
#20	Low	*433*
#28	Low	*1651*

Supplementary Table S3. Glioblastoma Patient Clinical Information.

Patient No	Gender	RT Received	TMZ Received	Survival (Days)
1	F	Y	Y	498
2	F	N	Y	201
5	M	Y	Y	372
7	M	Y	Y	637
9	M	Y	Y	111
10	M	Y	Y	432
11	F	Y	Y	227
13	M	N	Y	461
14	M	Y	N	396
15	M	N	N	344
16	F	Y	N	790
18	F	N	N	235
21	M	N	Y	624
24	M	Y	Y	55
25	F	Y	Y	427
26	M	Y	N	135
28	F	N	N	442
29	M	Y	N	537
30	M	Y	Y	310
31	M	N	N	735
32	M	Y	N	142
33	M	Y	Y	548
34	F	Y	Y	179
35	M	Y	Y	930
37	M	Y	Y	856
38	F	Y	Y	285
40	F	N	N	246
41	M	N	N	453
43	M	Y	N	435
45	M	Y	Y	442
49	F	Y	Y	275
51	F	Y	Y	401
52	M	Y	N	462
53	F	Y	N	510
54	M	Y	Y	593
55	F	Y	Y	485
56	M	Y	N	323
57	M	N	N	214

58	M	Y	N	433
59	F	Y	Y	394
62	M	N	N	419
63	M	Y	Y	515
65	M	Y	Y	480
66	F	Y	Y	231
67	M	N	N	120
68	M	N	Y	484
69	M	Y	N	921
70	M	Y	Y	402
71	F	Y	Y	379
72	F	N	N	79
73	M	Y	Y	375
78	M	Y	Y	498
79	M	Y	Y	295
80	M	Y	Y	1659
81	M	Y	N	148
82	M	Y	Y	962
85	M	Y	Y	895
87	M	Y	Y	655
89	F	Y	Y	23
93	F	Y	Y	571
95	F	N	N	396
96	M	Y	Y	349
98	M	Y	N	233
100	M	N	N	228
101	M	Y	N	902
102	M	N	N	88
103	F	Y	Y	934
104	F	Y	N	963
105	F	Y	Y	258
106	M	N	N	76
107	F	Y	Y	337
112	M	Y	Y	347
116	M	Y	Y	224
119	F	Y	Y	371
120	F	Y	Y	236
122	F	Y	Y	589
123	F	Y	Y	466
125	F	Y	Y	726
127	F	Y	Y	351
128	M	N	N	142

129	F	Y	Y	889
130	M	N	N	53
131	M	Y	Y	567
132	M	Y	Y	131
134	M	Y	Y	176
135	F	Y	Y	363
136	M	Y	Y	183
137	F	Y	Y	362
138	F	N	N	133
140	M	Y	Y	507
141	F	Y	Y	417
143	M	Y	Y	722
144	F	Y	Y	481
145	M	Y	Y	371
146	M	Y	Y	360
148	F	Y	Y	738
150	M	Y	Y	594
151	F	Y	Y	374
152	F	Y	Y	501
154	M	Y	Y	515
155	M	Y	Y	464
158	F	Y	Y	391

RT = Radiotherapy; TMZ = Temozolomide

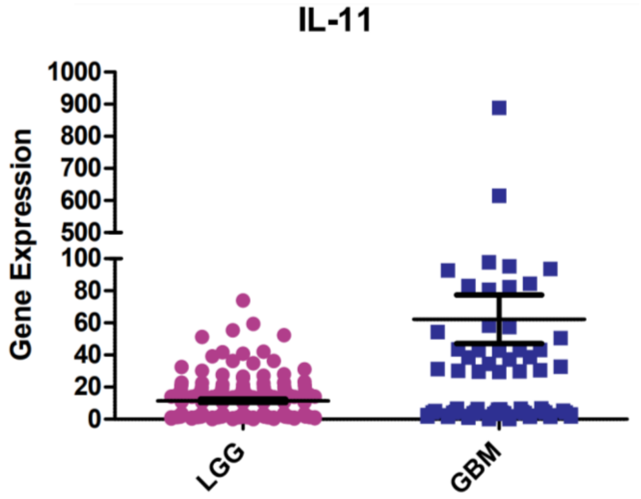

Supplementary Figure S1: The relationship between IL-11 gene expression in low grade glioma (LGG; Pink) and glioblastoma (GBM; Blue) glioma was determined through mining a SurvExpress TCGA dataset. (n=76, mean ± SD, where p<0.0001).

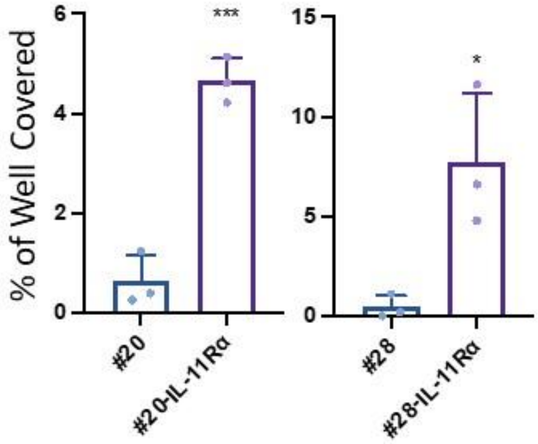

Supplementary Figure S2: #20, #20-IL-11Rα, #28 and #28-IL-11Rα cells were cultured in glucose-free RPMI media ± Glutamine and evaluated for survival using the cell viability assay (n=3, mean ± SD, where * indicates $p<0.05$ and *** indicates $p<0.001$).

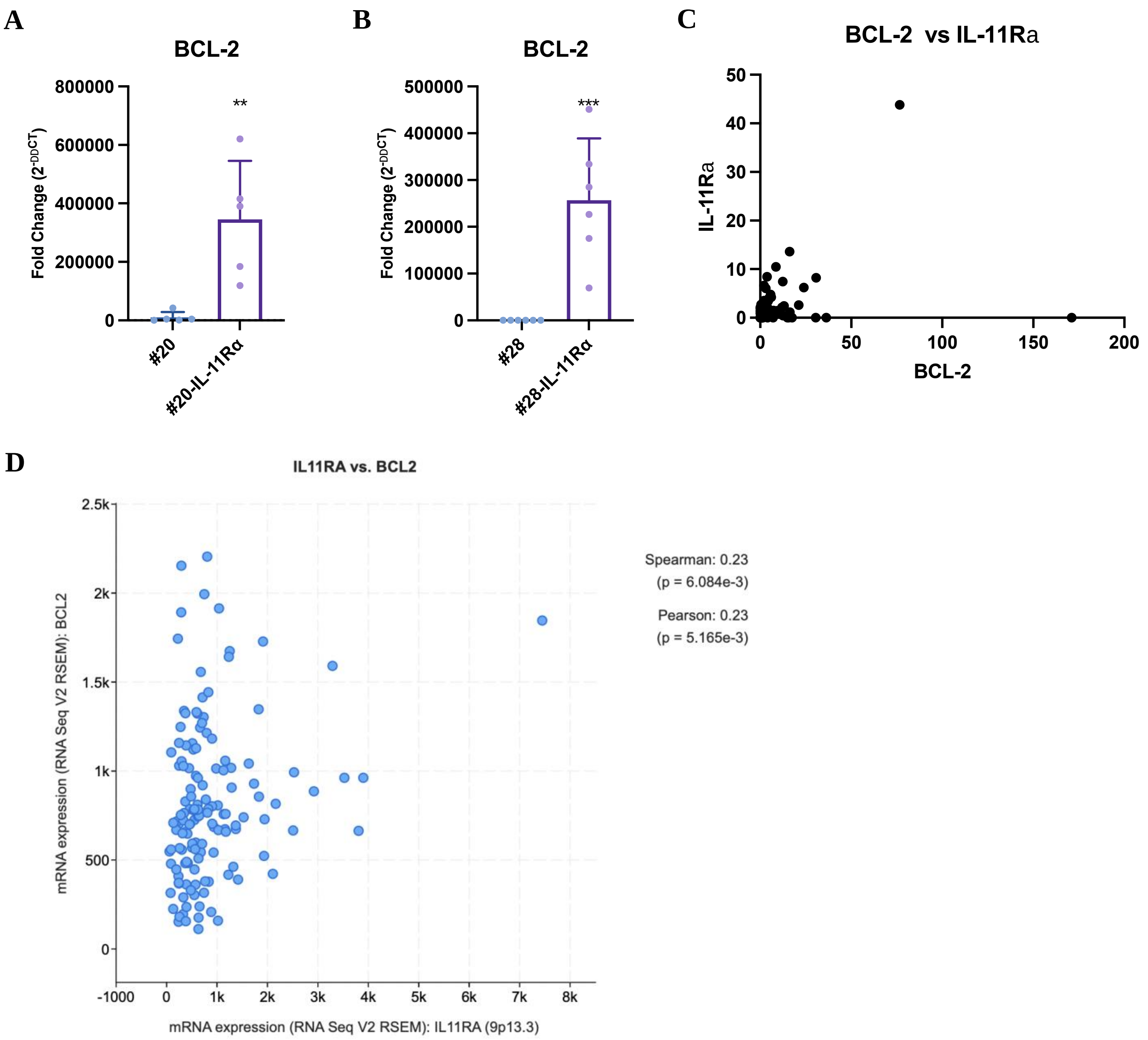

Supplementary Figure S3: Gene expression levels of the anti-apoptotic gene Bcl-2 were determined in **A.** #20 versus #20-IL-11Rα, **B.** #28 versus #28-IL-11Rα cells (n=3, mean ± SD, where ** indicates p<0.01 and *** indicates p<0.001) **C.** in our cohort of glioblastoma patient tumor tissue (n=75, where p=0.005 and R=0.3236). Il-11R expression significantly correlated with Bcl-2 gene expression. **D.** The TCGA where Il-11R expression significantly correlated with Bcl-2 gene expression.

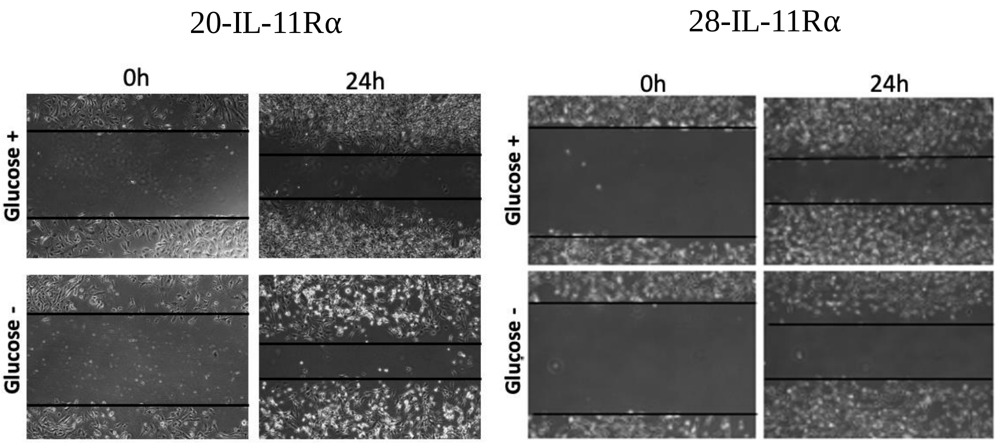

Supplementary Figure S4: #20-IL-11R*α* or #28-IL-11R*α* were grown to confluency then "wounded" at time 0 h. Cells were cultured in DME media containing glutamine ± glucose for a subsequent 24 h. Images of wound healing were taken at 0 and 24.
